# Large Scale 3D Morphable Models

**DOI:** 10.1007/s11263-017-1009-7

**Published:** 2017-04-08

**Authors:** James Booth, Anastasios Roussos, Allan Ponniah, David Dunaway, Stefanos Zafeiriou

**Affiliations:** 1 Imperial College London, London, UK; 2 Great Ormond Street Hospital, London, UK; 3 University of Exeter, Exeter, UK

**Keywords:** 3D morphable models, Dense correspondence, Demographic-specific models

## Abstract

We present large scale facial model (LSFM)—a 3D Morphable Model (3DMM) automatically constructed from 9663 distinct facial identities. To the best of our knowledge LSFM is the largest-scale Morphable Model ever constructed, containing statistical information from a huge variety of the human population. To build such a large model we introduce a novel fully automated and robust Morphable Model construction pipeline, informed by an evaluation of state-of-the-art dense correspondence techniques. The dataset that LSFM is trained on includes rich demographic information about each subject, allowing for the construction of not only a global 3DMM model but also models tailored for specific age, gender or ethnicity groups. We utilize the proposed model to perform age classification from 3D shape alone and to reconstruct noisy out-of-sample data in the low-dimensional model space. Furthermore, we perform a systematic analysis of the constructed 3DMM models that showcases their quality and descriptive power. The presented extensive qualitative and quantitative evaluations reveal that the proposed 3DMM achieves state-of-the-art results, outperforming existing models by a large margin. Finally, for the benefit of the research community, we make publicly available the source code of the proposed automatic 3DMM construction pipeline, as well as the constructed global 3DMM and a variety of bespoke models tailored by age, gender and ethnicity.

## Introduction

**Fig. 1:**
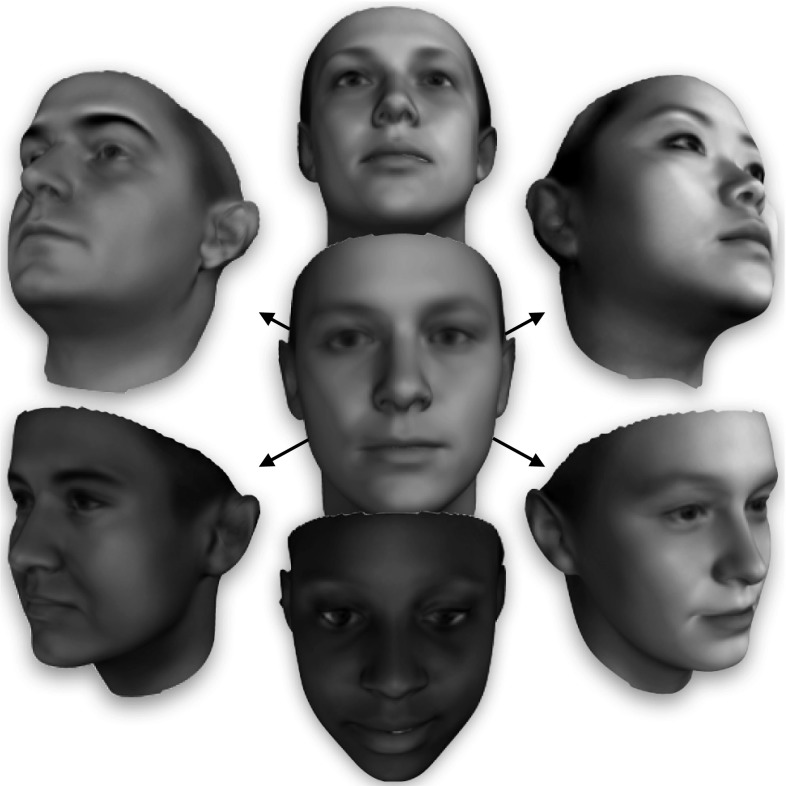
The sheer number of facial meshes used in training LSFM produces a 3D Morphable Model with an unprecedented range of human identity in a compact linear model

3D Morphable Models (3DMMs) are powerful 3D statistical models of the shape and texture of the human face.^1^ In the original formulation, as presented by the seminal work of  Blanz and Vetter (1999), a 3DMM used in an analysis-by-synthesis framework was shown to be capable of inferring a full 3D facial surface from a single image of a person. 3DMMs have since been widely applied in numerous areas in computer vision, human behavioral analysis, computer graphics, craniofacial surgery and large-scale facial phenotyping (Blanz and Vetter 2003, Amberg et al. 2008, Aldrian and Smith 2013, Staal et al. 2015, Hammond and Suttie 2012) (Fig. 1).

A 3DMM is constructed by performing some form of dimensionality reduction, typically principal component analysis (PCA), on a training set of facial meshes. This is feasible if and only if each mesh is first re-parametrised into a consistent form where the number of vertices, the triangulation, and the anatomical meaning of each vertex are made consistent across all meshes. For example, if the vertex with index *i* in one mesh corresponds to the nose tip it is required that the vertex with the same index in every mesh correspond to the nose tip too. Meshes satisfying the above properties are said to be in dense correspondence with one another. Whilst this correspondence problem is easy to state, it is challenging to solve accurately and robustly between highly variable facial meshes. Worst still, the very definition of anatomical meaning can be challenging to define for smooth regions of the face like the forehead or cheek, making objective measurement of correspondence quality difficult.

**Fig. 2:**
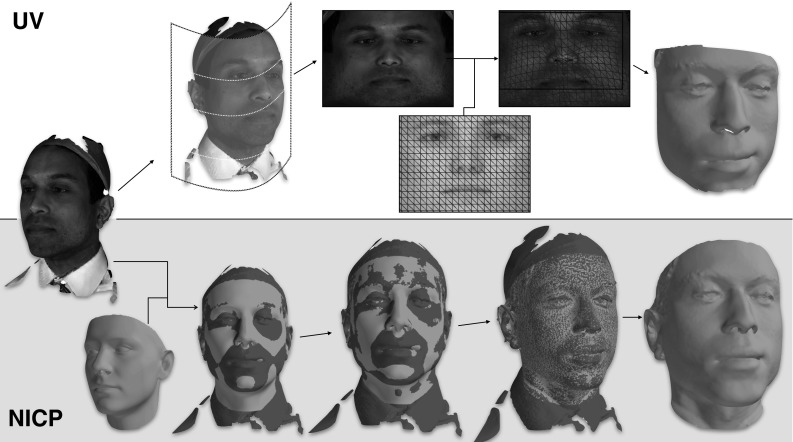
There are two techniques used to establish dense correspondence in 3DMMs. *Top* correspondence is established in a UV space—typically a cylindrical projection of the mesh shape and texture information. The UV image of each mesh is registered to a template UV image, and subsequent sampling produces a mesh in correspondence with the template. *Bottom* non-rigid iterative closest point (NICP), guided by sparse annotations, can be employed to iteratively deform a 3D template to match each mesh, avoiding the UV space entirely

Once built, 3DMMs provide two functions. Firstly, 3DMMs are powerful priors on 3D face shape and texture that can be leveraged in fitting algorithms to reconstruct accurate and complete 3D representations of faces from data deficient sources like in-the-wild 2D images or noisy 3D depth scan data. Secondly, 3DMMs provide a mechanism to encode any 3D face in a low dimensional feature space, a compact representation that makes tractable many 3D facial analysis problems.

### A Note on Terminology

For the sake of clarity, we note that here we explicitly define a *3D Morphable Model* as a statistical basis of shape and texture. A 3DMM is a data structure—a flexible representation of the 3D human face that can be persisted to disk and reused in a number of different contexts, both 2D and 3D in nature. We make this note as there is some flexibility in the literature as to whether a 3DMM refers to a statistical model, (a data structure, the view we take), or an *algorithm* for performing 3D reconstruction from a single image.

This confusion arrises from the fact that, as previously mentioned, the initial application of such models was in this one narrow application. However the usages of these models have expanded massively into new fields over the last 15 years. With emerging applications such as virtual reality (VR), autonomous vehicles, and depth-camera equipped consumer robotics, it is not hard to image a future where 3D applications of 3DMMs are more obvious and widespread than the initial application to 2D images. With this forward looking view, in this paper we are concerned with constructing a reusable statistical models that may be used in a myriad of applications.

### The Challenges of Large-Scale 3DMMs

In this paper we revisit 3DMMs under a new context—that we have access to a database of around 10,000 high quality 3D facial scans, with a wide variation of age, gender, and ethnicity represented amongst the subjects. Furthermore, for each individual we have detailed demographics including the subject’s age, gender, and ethnic background. Our goal is to leverage this data in order to build an anatomically accurate 3D Morphable Model that can be used in a wide variety of applications. This context brings with it a number of new challenges for 3DMM construction.

Firstly, the sheer scale of the data takes into uncharted territory. As we will motivate in Sect. 2, previous works have only worked with smaller datasets (generally two orders of magnitude smaller), where it is tractable to perform manual work in preprocessing meshes as part of the construction process. Furthermore, construction algorithms in the past have only been proven on datasets containing small variation in age and ethnicity (typically, dominated by adult caucasian subjects).

Secondly, we maintain a tight focus on producing an *anatomically accurate* 3D Morphable Model—by this we mean that the dense correspondence we seek to establish should optimally reflect the underlying anatomical structure of the human face. This means we actively avoid any alignment based on ‘skin-deep’ facial features, perhaps the most obvious of which would be eyebrows, as aligning such features would disrupt the alignment of the underlying facial structure. This is a subtle but important distinction. Perusing this goal opens up the use of 3DMM in applications where an accurate model of the underlying facial structure is key like craniofacial surgery planning and assessment.

Finally, we have wholly additional per-subject information in the form of detailed demographics, which opens up many new avenues of possibilities in both the construction and fitting of 3DMMs. Indeed, we will show for the first time clear evidence that the manifold of plausible faces is naturally clustered by demographics like age and ethnicity, and use this insight to devise new approaches to 3DMM construction and fitting that advance on the state of art. We further demonstrate for the first time that a large scale model coupled with accurate demographics enables accurate age classification from 3D shape data alone (Fig. 2).

### Paper Structure

The remainder of the paper is structured as follows. In Sect. 2 an overview of the Morphable Model construction literature will be given, whilst Sect. 3 will provide an overview of the contributions this paper makes to the field. Section 4 provides a mathematical framework for 3DMM construction. The most challenging and varied component of construction, establishing dense correspondence, will get its own treatment in Sect. 5, where we will describe and analyze in detail three popular approaches to solving this problem in our specific context.

Informed from this work, Sect. 6 will put forward our novel pipeline for automated anatomically accurate 3D Morphable Model construction. In Sect. 7 we will evaluate this pipeline by applying it to the newly-introduced MeIn3D dataset, to construct large scale facial model (LSFM). We examine in detail the properties of this unique model, and test its performance in a range of applications including age prediction and 3D model fitting. Finally, Sect. 8 will provide some conclusions and ideas for future work in this area.

## Previous Work

The construction of a 3DMM usually consists of two main steps—establishing group-wise dense correspondence between a training set of facial meshes, and then performing some kind of statistical analysis on the registered data to produce a low-dimensional model.

In the original formulation, Blanz and Vetter (1999) solved the dense correspondence problem by representing each facial mesh in a cylindrical ‘UV’ map, flattening each 3D surface down into a 2D space. This reduced establishing correspondence to a well-understood image registration problem, which was solved with a regularized form of optical flow. Blanz and Vetter employed PCA to construct their model, and showed that in their framework, model performance was improved by segmenting the facial surface into regions (eyes, nose, mouth, other), building individual models per-component, before blending resulting segments back together. Amberg et al. (2008) extended this approach to emotive facial shapes by adopting an additional PCA modeling of the offsets from the neutral pose. This resulted to a single linear model of both identity and expression variation of 3D facial shape.

Blanz and Vetter’s correspondence technique was only used to align the facial meshes of 200 subjects of a similar ethnicity and age (Blanz and Vetter 1999). This approach was effective in such a constrained setting, but it is fragile to large variance in facial identity. To overcome this limitation, Patel and Smith (2009) proposed to manually annotate the cylindrical face projections with a set of sparse annotations, employing a thin plate splines (TPS) warp (Bookstein 1989) to register the UV images of the meshes into a common reference frame. Cosker et al. (2011) automated the procedure of landmark annotations required for the TPS warp, for the special case of temporal sequences of a single identity displaying emotions. Several facial landmarks on a handful of meshes for a given temporal sequence were manually annotated and used to build a person-specific active appearance model (AAM) (Cootes et al. 2001) that was then used to automatically find sparse annotations for each frame in the data set.

As an alternative to performing alignment in a UV space, Paysan et al. (2009a) built the basel face model (BFM) by using an optimal step nonrigid ICP algorithm (Amberg et al. 2007) (NICP) to directly align scans of 200 subjects with a template. This native 3D approach was guided by manually placed landmarks to ensure good convergence.

Brunton et al. (2011) adopt wavelet bases to model independent prior distributions at multiple scales for the 3D facial shape. This offers a natural way to represent and combine localized shape variations in different facial areas.

Vlasic et al. (2005) modeled the combined effect of identity and expression variation on the facial shape by using a multilinear model. More recently, Bolkart and Wuhrer (2015) show how such a multilinear model can be estimated directly from the training 3D scans by a joint optimization over the model parameters and the groupwise registration of the 3D scans.

For the case where a temporal sequence of meshes is available, Bolkart and Wuhrer (2015) fit a multilinear model and estimate a 4D sequence parametrization. This can be used to animate a single 3D scan with a specific facial expression. Another alternative to modeling emotive faces is the blendshape model, which was used by Salazar et al. (2014) to place into correspondence emotive faces in a fully automated way. For more details on 3D facial shape modeling, we refer the interested reader to the recent extensive review article of Brunton et al. (2014b) and the references therein.

Due to the costly manual effort currently required to construct 3DMMs from 3D data, recent efforts in the field have also focused on trying to build models from other data sources. Kemelmacher-Shlizerman (2013) recently presented a technique that attempts to learn a full 3D facial model automatically from thousands of images. Whilst impressive given the input data, such techniques cannot currently hope to produce models comparable in resolution and detail to techniques that natively process 3D input data.

All the aforementioned works do not use more than 300 training facial scans. In this paper we show that such a size of training set is far from adequate to describe the full variability of human faces. On top of that, all existing works use training sets with a very limited diversity in the ethnic origin (mostly European/Caucasian) as well as in the age (mostly young and middle adulthood) of the subjects.

Due to this kind of limitations of the training sets adopted, no existing work so far, to the best of our knowledge, has developed demographically-specific 3DMM models, i.e. 3DMM models tailored for specific age, gender or ethnicity groups. The above issues pose severe limitations in the descriptive power of the resultant Morphable Models.

At the same time, there is strong experimental evidence that the 3D facial shapes of disparate gender and ethnicity are significantly separable. Toderici et al. (2010) perform an accurate estimation of gender and ethnicity based purely on the 3D facial shapes, without using any associated texture or photographic information. Their proposed method achieves around 99% accuracy for race and 94% for gender recognition.

It is also evident from the prior art that demographically-specific modelling is able to achieve substantial improvements on 3D face recognition performance. Heo and Savvides (2012) use demographically-specific models in the case of generic elastic modelling (GEM), which is a much coarser modelling of 3D shape variation than 3DMMs. The authors are solely based on 2D training images and a depth-based representation of facial variation. In their extensive experimental evaluation, they show that the demographically-specific models achieve significantly better 3D reconstruction as well as face recognition performance across different views, as compared to the corresponding global models.

There currently exists only three publicly available 3D Morphable Models. Firstly, a University of Basel website provides the BFM model (Paysan et al. 2009a).^2^ Secondly, Bolkart, Brunton, Salazar and Wuhrer have a website where they provide 3DMMs constructed by their recent works, modelling 3D face shapes of different subjects in neutral expression (Brunton et al. 2014b) as well as 3D shapes of different subjects in different expressions (Brunton et al. 2014a, Bolkart and Wuhrer 2015).^3^ Finally, a University of Surrey website provides a range of 3D facial shape models of varying resolutions (Huber et al. 2016).^4^

## Contributions

Our goal is to make it trivial to build 3D Morphable Models automatically from large collections of 3D scans. We believe that our automated pipeline significantly lowers the barrier to entry for facial Morphable Model construction, to the point where there is no need to choose a trade off between automation and model quality. We are able to do this by capitalising on the powerful, person independent, facial landmark localisation frameworks that have been recently introduced (Alabort-i Medina et al. 2014).

Our contributions in this paper are three fold.

Firstly, we quantitatively compare the three most popular techniques for establishing dense correspondence in 3DMM construction—NICP, and two UV based interpolations, UV-TPS and UV-optical flow (UV-OF). We perform this analysis in the context of automatic model construction, the first time such an comparison has been presented to the community.

Secondly, informed by our in-depth comparison of dense correspondence methods, we introduce a novel robust pipeline for 3DMM construction that is completely automated. More precisely, we develop a novel and robust approach to 3D landmark localization, followed by dense correspondence estimation using the NICP algorithm. Then, we propose an approach to automatically detect and exclude the relatively few cases of failures of dense correspondence, followed by PCA to construct the deformation basis. We pay particular attention to the efficiency and scalability of all the aforementioned steps. We make the source code of this pipeline publicly available, for the benefit of the community.^5^

Finally, we use our pipeline on a 3D facial database of 9663 subjects to construct LSFM, the largest and most information-rich 3DMM of face shapes in neutral expression produced to date.

LSFM is built from two orders of magnitude more identity variation than current state-of-the-art models. We conduct extensive experimental evaluations that show that this additional training data leads to significant improvements in the characteristics of our 3D Morphable Model, and demonstrate that LSFM outperforms existing models by a wide margin. We also present experiments that study the effect of using larger datasets and more varied demographics in model construction. These experiments provide for the first time a comprehensive answer to the question of how much training data is needed for 3DMMs before effects of diminishing returns set in.

Apart from building LSFM using the commonly-used global PCA, we also build a collection of PCA models tailored by age, gender and ethnicity, capitalizing on the rich demographic information of the used database. We present quantitative experimental evidence of why and when such tailored models should be preferred over the global PCA.

Using the demographic information, we are also able to analyze for the first time the distribution of faces on the low-dimensional manifold produced by the global PCA. We visualize the manifold of faces using t-distributed stochastic neighbor embedding (t-SNE) (Maaten and Hinton 2008), and report on clear age and ethnic clustering that can be observed. As an application example, we utilize the proposed model to perform age classification, achieving particularly accurate results.

The global LSFM model as well as the models broken down by demographics will be made publicly available from this work.^6^ It is worth mentioning that current progress in computer vision would not be possible without the collection of large and comprehensive datasets e.g. Everingham et al. (2010), Sagonas et al. (2013), Jain and Learned-Miller (2010), Deng et al. (2009), and we believe that our publicly available models contributes towards this effort.

## Background

### Data Representation

The geometry of a 3D facial mesh is defined by the vector $\mathbf {X}= {[\mathbf {x}_1^T,\mathbf {x}_2^T,\ldots ,\mathbf {x}_n^T]}^T \in \mathbb {R}^{3n}$, where $n$ is the number of vertices and $\mathbf {x}_i = {[x_x^i,x_y^i,x_z^i]}^T \in \mathbb {R}^3$ describes the X,Y and Z coordinates of the *i*-th vertex.

The topology of a mesh is encoded in a triangle list $\mathbf {T}= [\mathbf {t}_1^T,\mathbf {t}_2^T,\ldots ,\mathbf {t}_m^T]\in \mathbb {R}^{3\times m}$, where $m$ is the number of triangles and $\mathbf {t}_i = [t_1^i,t_2^i,t_3^i]$ is the index triplet that defines the *i*-th triangle. Note that the indices $\;t_j^i \in \{\mathbb {Z^+}\; | \; t_j^i \le n\}$ correspond to vertices of the mesh.

Texture information is given as a per-vertex color vector $\mathbf {C}= {[c_1^T, c_2^T, \ldots , c_n^T]}^T$ where $c_i = [R_i, G_i, B_i] \in \mathbb {R}^3$.

A triangle mesh $\mathbf {M}= \{\mathbf {X}, \mathbf {T}\}$ is thus comprised of $n$ vertices and $m$ triangles. If the mesh is textured, the definition is augmented to include the per vertex color information: $\mathbf {M_t}= \{\mathbf {X}, \mathbf {T}, \mathbf {C}\}$.

**Fig. 3:**
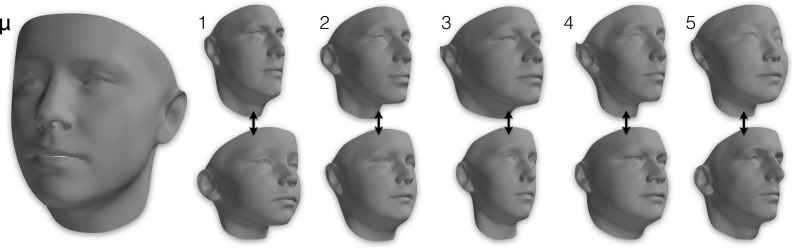
Visualisation of the shape model of LSFM-global: mean shape ($\mu$) and first five principal components, each visualized as additions and subtractions away from the mean shape. In more detail, the *top* (*bottom*) row corresponds to deviating from $\mu$ in the direction of the corresponding shape eigenvector, with a weight of 3$\sigma _i$ ($-3\sigma _i$), where $\sigma _i$ is the standard deviation of the corresponding component

### 3D Face Database Overview

The collected database, which we refer to as *MeIn3D*, contains approximately 12,000 3D facial scans captured over a period of 4 months. A 3dMD™ photometric stereo capture device was utilized, creating a 3D triangular surface for each subject composed of approximately 60,000 vertices joined into approximately 120,000 triangles, along with a high resolution texture map. Furthermore, 9663 subjects also provided metadata about themselves, including their gender, age and ethnicity. This information allows for the construction of models for targeted populations, such as within a defined age range or from a particular ethnic background. The dataset covers a wide variety of age (see Fig. 7), gender ($48\%$ male, $52\%$ female), and ethnicity ($82\%$ White, $9\%$ Asian, $5\%$ mixed heritage, $3\%$ Black and $1\%$ other) (Fig. 3).

### 3DMM Construction

The input to a 3DMM construction algorithm is a set of $k$ meshes $\{\mathbf {M}_1,\mathbf {M}_2,\ldots ,\mathbf {M}_k\}$. Each input mesh has its own number of vertices and triangles, and a particular ordering to its topology.

The construction of a 3DMM happens in two distinct stages. First, a state of dense correspondence needs to be established between the training set meshes. Following this, a statistical analysis step on the correspondng meshes yields linear models of shape and texture.

#### Dense Correspondence

In this procedure, a collection of meshes are re-parameterized into a form where each mesh has the same number of vertices joined into a triangulation that is shared across all meshes. Furthermore, the semantic or anatomical meaning of each vertex is shared across the collection. Meshes satisfying the above conditions are said to be in dense correspondence with one another. Given such a collection of meshes, the dense correspondences among them are typically found through the registration of every mesh with a template. Landmark annotations are used as additional priors that guide the registration process in the corresponding sparse locations. Dense correspondence can be seen as a generalization of non-rigid image registration to triangular meshes.

Of particular interest to us in 3DMM construction is the case where multiple meshes share the same topology $\mathbf {T}$, which as we will see is a necessary consequence of meshes being in dense correspondence. In such cases we can dispense with concerning ourselves with the mathematically clumsy definition of $\mathbf {M}$ and directly work with the vectors of shape $\mathbf {X}$ and texture $\mathbf {C}$, bearing in mind that we assume an implicit shared triangulation $\mathbf {T}$.

Note that we will explore mechanisms for establishing dense correspondence in some depth in Sect. 5.

#### Similarity Alignment and Statistical Modelling

Given a set of meshes in dense correspondence, we now wish to build a statistical model of shape and texture.

The collection of meshes in dense correspondence are subjected to Procrustes Analysis to remove similarity effects, leaving only shape information. The processed meshes are statistically analysed, typically with principal component analysis (Davies et al. 2008), generating a 3D deformable model as a linear basis of shapes. This allows for the generation of novel shape instances:

1 $$\begin{aligned} \mathbf {X}^{*} = \bar{\mathbf {X}} + \sum _{i=1}^{k_{\varvec{\alpha }}} \varvec{\alpha }_i \mathbf {U}_i = \bar{\mathbf {X}} + \mathbf {U}\mathbf {\varvec{\alpha }} \end{aligned}$$

where $\bar{\mathbf {X}} \in \mathbb {R}^{3n}$ is the mean shape and $\mathbf {U}=[\varvec{U}_1 \ldots \varvec{U}_d]\in \mathbb {R}^{3n\times d}$ is the orthonormal basis matrix whose columns contain the shape eigenvectors $\varvec{U}_i$. Also, $\varvec{\alpha }=[\alpha _1,\ldots ,\alpha _d]\in \mathbb {R}^{d}$ is the shape vector that contains the parameters (coefficients) $\alpha _d$ that define a specific shape instance under the given deformable model. The degrees of freedom of this model are given by the number of principal components *d*, which is much smaller than the dimensionality 3*n* of the original space of 3D shapes. Note that this model is combined with the fixed triangle topology that was yielded from the stage of dense correspondences estimation.

Interpolating color values from nearby vertices with a barycentric weighting allows for the construction of a orthonormal texture model with the same formulation as above:

2 $$\begin{aligned} \mathbf {C}^* = \bar{\mathbf {C}} + \sum _{i=1}^{k_\beta } \beta _i \mathbf {C}_i = \bar{\mathbf {C}} + \mathbf {V}\mathbf {\beta } \end{aligned}$$

where $\bar{\mathbf {C}} \in \mathbb {R}^{3n}$ is the mean texture sample and $\mathbf {V}=[\varvec{V}_1 \ldots \varvec{V}_d]\in \mathbb {R}^{3n\times d}$ is the orthonormal basis matrix whose columns contain the texture eigenvectors $\varvec{V}_i$. Also, $\varvec{\beta }=[\beta _1,\ldots ,\beta _d]\in \mathbb {R}^{d}$

**Fig. 4:**
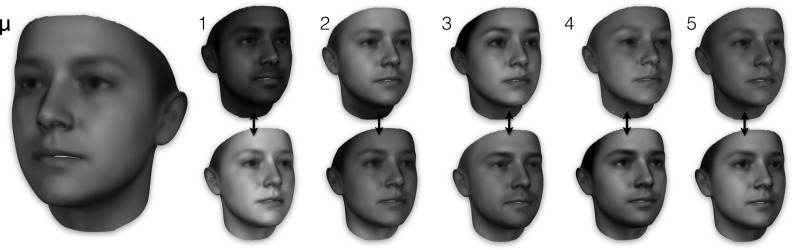
Visualisation of the texture model of LSFM-global: mean texture ($\mu$) and first five principal components, each visualized as additions and subtractions away from the mean texture. In more detail, the *top* (*bottom*) row corresponds to deviating from $\mu$ in the direction of the corresponding texture eigenvector, with a weight of 3$\sigma _i$ ($-3\sigma _i$), where $\sigma _i$ is the standard deviation of the corresponding component. All textures are visualized on the mean 3D shape

Any input 3D mesh $\mathbf {X}$ can be projected on the model subspace by finding the shape vector $\varvec{\alpha }$ that generates a shape instance Eq. (1) that is as close as possible to $\mathbf {X}$. The optimum shape vector and the corresponding projection $P(\mathbf {X})$ on the model subspace are given by Davies et al. (2008):

3 $$\begin{aligned} \varvec{\alpha } = \mathbf {U}^T (\mathbf {X}- \bar{\mathbf {X}}),\quad P(\mathbf {X}) = \bar{\mathbf {X}} + \mathbf {U}\mathbf {U}^T (\mathbf {X}- \bar{\mathbf {X}}) \end{aligned}$$

## Dense Correspondence Approaches

Having now considered an overview of how 3D Morphable Models are constructed we focus in on the most challenging and variable aspect of the procedure—establishing dense correspondence.

All dense correspondence algorithms typically take as input a template and a target mesh that have been landmarked with sparse annotations. Establishing dense correspondence can thus be thought of as an interpolation problem; a known correspondence for a small subset of vertices needs to be extended to all vertices in the template. In Sect. 6.1 we explain our novel approach for automatically finding annotations, for now we assume landmarks can be reliably found and examine the dense correspondence methods in isolation.

### UV-Space-Based Dense Correspondences

The first technique proposed for establishing dense correspondence in 3DMM construction defined a 2D ‘UV’ space for each mesh—a contiguous flattened atlas in which the 3D surface of the face can be embedded (see top of Fig. 2). Such a UV space is associated with its corresponding 3D surface through a bijective mapping, and so it follows that establishing dense correspondence between two UV images implicitly establishes a 3D-to-3D correspondence for the mapped mesh. The key assumption in this case is that it is possible to create UV mappings that accurately represent the 3D facial surfaces. This technique is popular as it reduces the challenging 3D correspondence problem to a well-studied 2D image non-rigid alignment one. It also may be seen as the most natural way to register laser scanned 3D meshes as it takes place in the native domain of the scanning device. For other 3D capture devices, Booth and Zafeiriou (2014) outlined how a UV style space can be synthetically created from the raw capture data through simple spherical or cylindrical projections of the data. Each UV map is an image—each pixel encoding both spatial information (X, Y, Z) and texture information (R, G, B).

UV-space-based dense correspondence techniques apply a non-rigid image alignment between all UV maps of the meshes and a reference UV map, registering all UV maps into a consistent reference space. A consistent sampling of each aligned UV space is then performed. At each sampling site, a vertex is created by sampling from the corresponding spatial information. Texture information can either be extracted densely per-pixel, (so a single RGB colour value is assigned per vertex) or a texture coordinate into the texture UV map can be assigned (so the texture mapping can be of a much higher density than the spatial mapping). In our treatment we will always use the simpler per-vertex color sampling, but we note it is trivial to change this, with the benefit of allowing shape and texture models to be of differing resolutions.

Since the UV space representation is effectively a 2D image representation, each UV map of the database can be aligned with the reference UV map by applying an image registration algorithm. Usually, one of the following two approaches are adopted for this task:

- Thin plate splines (TPS) interpolation, as e.g. done in Patel and Smith (2009).
- Optical flow (OF) estimation, as e.g. done in Blanz and Vetter (1999).

We refer to the corresponding dense correspondence techniques as UV-TPS and UV-OF respectively.

In UV-TPS, a dense mapping between the UV maps is estimated via a TPS interpolation of the correspondences that are established by the sparse landmark annotations. In UV-OF, each pair of UV maps is registered by applying optical flow on the multichannel image data defined on the UV space by the texture and the 3D cylindrical coordinates of the face points.

### Non-Rigid Iterative Closest Point (NICP)

In contrast to the UV-space-based approaches, Amberg et al. (2007) propose a natively 3D algorithm, which directly establishes 3D-to-3D correspondences. The algorithm of Amberg et al. (2007) extends the (rigid) ICP approaches to nonrigid deformations while retaining tractable convergence properties. It is based on a locally affine representation of the deformations and adopts an adjustable stiffness parameter.

In more detail, let $\mathbf {S}$ be the 3D shape of any of the 3D scans of the considered database. Note that each scan could have a different, arbitrary number of vertices. Also, let $\mathbf {V} \in \mathbb {R}^{3n}$ be the 3D mesh of the adopted facial template, where $n$ being the number of vertices of this template. The NICP method of Amberg et al. (2007) non-rigidly deforms the template $\mathbf {V}$ in order to match with the input 3D scan $\mathbf {S}$ as accurately as possible. This deformation is over-parametrised with a collection $\mathbf {A} = \{ \mathbf {A}_1,\ldots ,\mathbf {A}_n\}$ of affine transformations, one for each vertex of the template. Each $\mathbf {A}_i$ is a 3$\times$4 affine transformation matrix that is applied on the *i*-th vertex of the template $v_i\in \mathbb {R}^3$, resulting to the location of the vertex after the nonrigid deformation: $\hat{\varvec{v}}_i = \mathbf {A}_i (\varvec{v}_i^T , 1)^T$.

The deformation of $\mathbf {V}$ is based on the minimisation of the following energy:

4 $$\begin{aligned} E(\mathbf {A}) = E_d( \mathbf {A}) + \alpha E_s(\mathbf {A}) + \beta E_{\ell }(\mathbf {A}) \end{aligned}$$

where $\alpha$ and $\beta$ are positive weights that balance the importance of the different terms (Fig. 4).

$E_d( \mathbf {A})$ is a data term that penalises the distance between the deformed version of the template and the 3D scan $\mathbf {S}$:

5 $$\begin{aligned} E_d(\mathbf {A}) = \sum _{i=1}^{N_F} \texttt {dist}^2(\mathbf {A}_i (\varvec{v}_i^T , 1)^T , \mathbf {S}) \end{aligned}$$

where $\texttt {dist}(\hat{\varvec{v}}_i,\mathbf {S})$ is the distance between the point $\hat{\varvec{v}}_i$ and the mesh $\mathbf {S}$ (with this mesh being considered as a triangulated surface to effectively compute the point-to-mesh distance).

$E_s(\mathbf {A})$ is a stiffness term that acts as a spatial regularisation of the deformed surface, favoring spatially smooth deformations. It penalises the differences between affine transformations that are neighbours in the mesh structure:

6 $$\begin{aligned} E_s(\mathbf {A}) = \sum _{(i,j)\in \mathcal {E}} \Vert (\mathbf {A}_i - \mathbf {A}_j) G \Vert _F^2 \end{aligned}$$

where $\mathcal {E}$ is the set of edges of the template and $\Vert \cdot \Vert _F$ denotes the Frobenius norm. Also, $G=\texttt {diag}(1,1,1,\gamma )$ is a weighting matrix that makes this cost function be a weighted sum of squared differences, where $\gamma$ balances the importance between differences in the translational part of the deformations (last column of $\mathbf {A}_i$) and differences in their rotational and skew part (first 3 columns of $\mathbf {A}_i$).

Finally, $E_{\ell }(\mathbf {A})$ is a sparse landmarks term that ensures that the deformed template is in accordance with the landmark information on the 3D scan $\mathbf {S}$:

7 $$\begin{aligned} E_\ell (\mathbf {A}) = \sum _{i=1}^L \Vert \mathbf {A}_{k_i} (\varvec{v}_{k_i}^T , 1)^T - \varvec{\ell }_i \Vert ^2 \end{aligned}$$

where *L* is the number of landmarks and $\varvec{\ell }_i\in \mathbb {R}^3$ is the location of the *i*-th landmark in the 3D scan $\mathbf {S}$. Finally, $k_i$ is the vertex index of the *i*-th landmark, with respect to the mesh of the template.

The method of Amberg et al. (2007) proposes an efficient and accurate minimisation of the energy (4)—we invite the interested reader to explore this paper for more details.

**Fig. 5:**
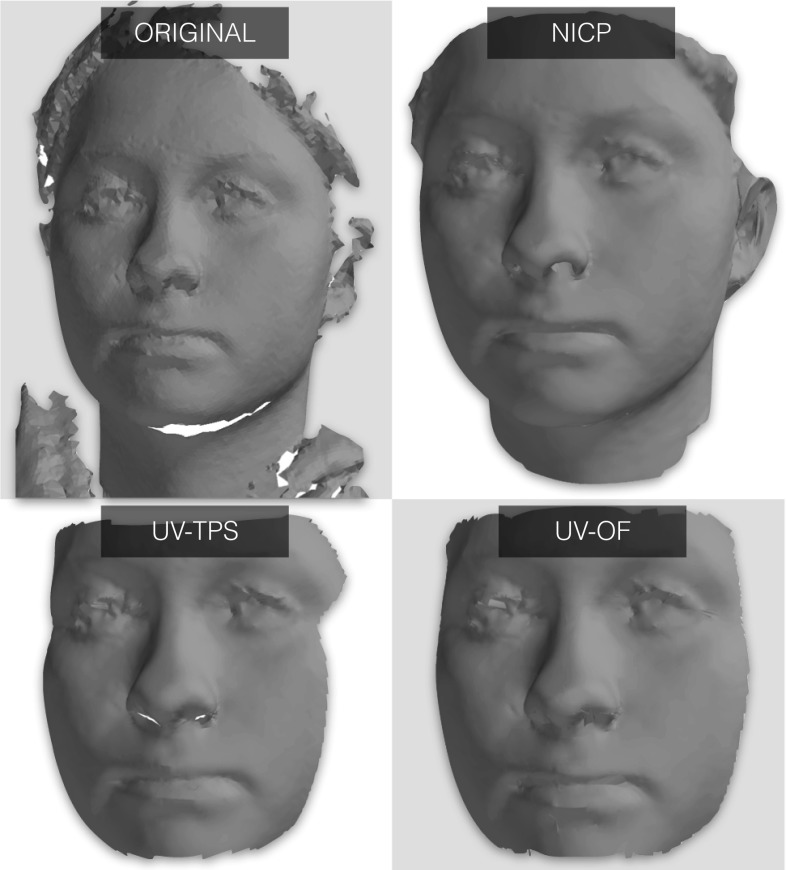
Example dense correspondence results from three techniques. NICP is better able to deal with parts of the face that don’t project to a cylindrical UV space well like the interior of the nose and mouth, and is less prone to drift effects

### Comparison of Dense Correspondence Approaches

Having described the two popular families of dense correspondence techniques, we now compare their traits, and motivate from a theoretical standpoint why we use NICP in our proposed pipeline. Empirical evidence supporting these thoughts will be provided in Sect. 7.6, where we will see how the different dense correspondence techniques impact the quality of the 3DMM we are able to construct from the MeIn3D dataset.

UV-based correspondence approaches are powerful in allowing for the simple reuse of image registration techniques, and are computationally efficient. As noted, they may also operate in the native domain for some 3D capture devices. However they are not without some disadvantages. Principally, a UV map can preclude the mapping of intricate details of face shape like the interior of the nostrils and the interior of the mouth. Furthermore, the UV space, which is typically a cylindrical projection of the 3D facial surface, introduces non-linearities into the dense correspondence process. For example, a uniform sampling in the UV space would lead to evenly sized triangles and evenly spaced vertices only in the case of a perfect cylinder. In areas of the face that differ greatly from this (such as the sides of the nose) the sampling will be no longer uniform. Furthermore, registering such cylindrical projections together can also introduce errors due to this same effect. In essence, we are relying on every face to share the same non-linearities to ‘cancel out’ each other to have a successful registration. When this is not the case (for instance there is a huge variation in nose shape) our registration must in some way be compromised by such issues (Fig. 5).

Finally, UV maps simply complicate the pipeline for 3DMM construction, in the sense that they require a rasterizing of the UV image and a subsequent sampling of the space to rebuild a mesh.

On the other hand, NICP is a principled energy minimization problem that avoids a number of these pitfalls. An argument against NICP would be that is an entirely geometry and topology-driven technique. The UV shape can in general admit shape and texture information, which can be used in driving the correspondence calculation (for instance, aligning similar skin pigment regions together). However, in our particular context, this behavior becomes somewhat of a liability for two reasons. Firstly, we again are seeking to find an anatomically relevant statistical model of the human face. Any texture information included my bias the dense correspondence calculation, compromising the quality of the model. Secondly, we again point out that MeIn3D contains a huge variety of ethnicity variation, which one could reasonably expect would affect the ability for techniques like optical flow to find good correspondences.

**Fig. 6:**
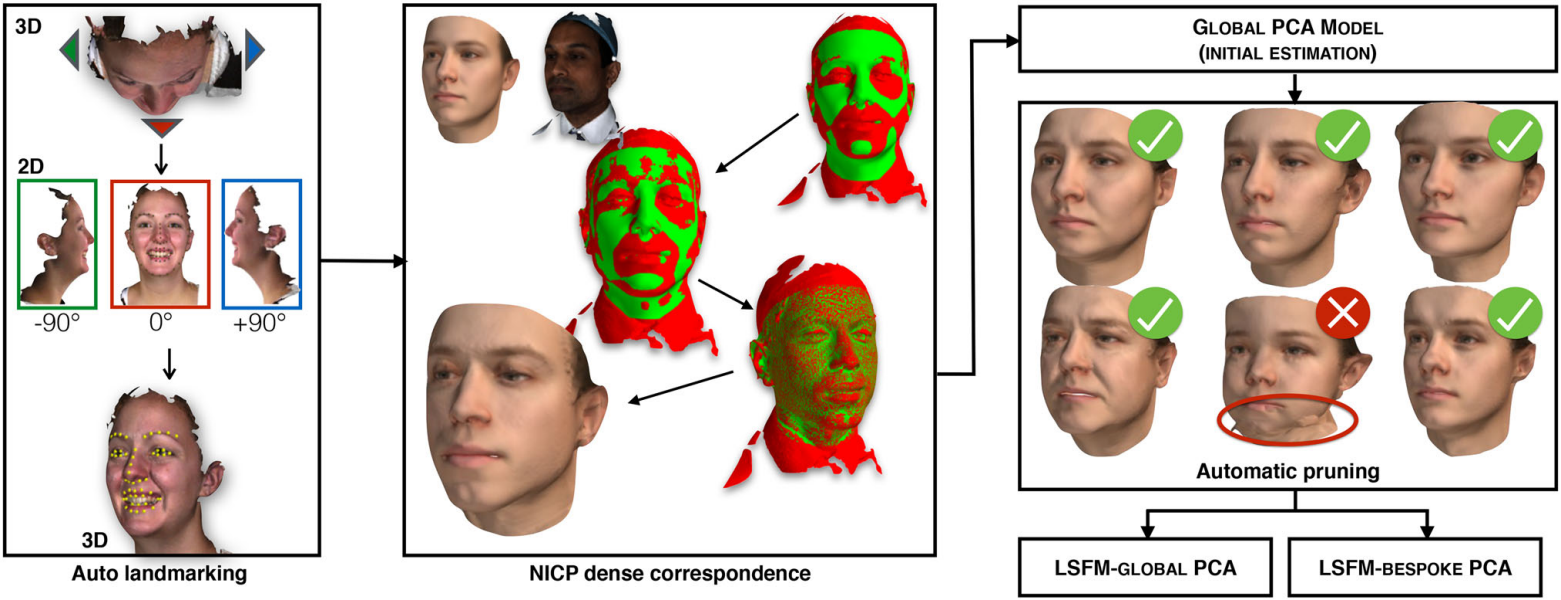
Our fully automated pipeline for constructing large scale 3DMMs. (1) Automatic landmarking based on synthetically rendered views. The rendered views have per-pixel shape information registered with them, and so the 2D landmarks can be projected reliably back to the 3D surface. (2) Guided by the automatic landmarks, the 3D template is iteratively deformed to exactly match every 3D facial mesh of the dataset. (3) A initial global PCA is constructed, and (4) erroneous correspondences are automatically removed. (5) LSFM models are constructed from the remaining clean data

## Proposed Pipeline

Let us consider the scenario that, as with MeIn3D database, one has a large-scale database of 3D facial scans and wants to apply a technique to construct a high-quality 3DMM. Such a large database raises some unique scalability challenges. We believe that it is highly beneficial to have a fully automated technique that would not require any kind of manual annotation. It is also very important that this technique is efficient in terms of both runtimes and memory requirements.

We introduce a 3DMM construction pipeline that meets all the aforementioned specifications, see Fig. 6. It starts with a novel and robust approach to 3D landmark localization. The 3D landmarks are then employed as soft constraints in NICP to place all meshes in correspondence with a template facial surface. With such a large cohort of data, there will be some convergence failures from either landmarking error or NICP. We propose a refinement post-processing step that weeds out problematic subjects automatically, guaranteeing that the LSFM models are only constructed from training data for which we have a high confidence of successful processing.

### Automatic Annotation

Image landmark localization is a well studied field. Our proposed technique allows us to bring to bear the huge expertise developed in image landmark localization to 3D landmark localization, allowing us to leverage the extensive datasets and state of the art techniques that are now readily available in this domain (Alabort-i Medina et al. 2014). This approach is similar to the work of Cosker et al. (2011) which was shown to be successful for temporal person-specific sequences, but here we pay particular attention to mesh sets with highly variable identity.

We do this by rendering each mesh from a number of virtual cameras positioned around the subject. Each virtual camera, which has a realistic perspective projection camera matrix, records an RGB texture image and an XYZ shape image. The texture view is a typical image of a face with a known pose, and so we are able to use a HOG active appearance model, a state-of-the-art image landmark localization technique (Antonakos et al. 2014), initialized from a state-of-the-art face detector (King 2009, Alabort-i Medina et al. 2014), in order to robustly locate a set of 68 sparse annotations in the view. The HOG AAM was trained on the diverse labelled face parts in the wild (LFPW) dataset (Belhumeur et al. 2011), and so is highly robust to pose, ethnicity, and emotive variation.

We train pose-specific landmark localization models for each view rendered, and use the shape images to project the fitting to the 3D surface, compositing the resulting 3D landmarks found into a master annotation set. Figure 6a graphically shows our landmark localisation technique.

### Dense Correspondences

Following the analysis in Sect. 5.3, and motivated by the empirical evidence we will put forward in Sect. 7.6, we choose to adopt the most effective correspondence approach, namely the NICP method. This method needs the specification of a template mesh and our choice is the mean face of the BFM model (Paysan et al. 2009a).

Each mesh is individually placed in correspondence with the template mesh. In more detail, we first use the automatically extracted landmarks to perform an optimal similarity alignment between the mesh in question and the (annotated) template, adopting Procrustes analysis. We then use NICP to deform the template so that it takes the shape of the input mesh, with the automatic landmarks acting as a soft constraint. The resulting deformed templates are re-parameterized versions of each subject which are correspondence with one another.

### Automatic Error Pruning

With such a large number of subjects there will be some failure cases at this stage. This is an unavoidable byproduct of the fact that both landmark localization and NICP are non-convex optimization problems that are sensitive to initialization. Our approach embraces this, seeking to weed out the small number of failure cases given the huge amount of data available for processing.

To remove outliers, we first construct an initial global PCA from all fittings. This PCA model of shape variation is expressed by Eq. (1). Adopting a commonly-used probabilistic interpretation of this model, we assume that the shape parameters $\alpha _1,\ldots ,\alpha _d$ are independent random variables and that each $\alpha _i$ follows a Gaussian distribution with zero mean and variance $\lambda _i$, where $\lambda _i$ is the *i*-th PCA eigenvalue (i.e. the *i*-th eigenvalue of the training data covariance matrix) (Davies et al. 2008).

**Fig. 7:**
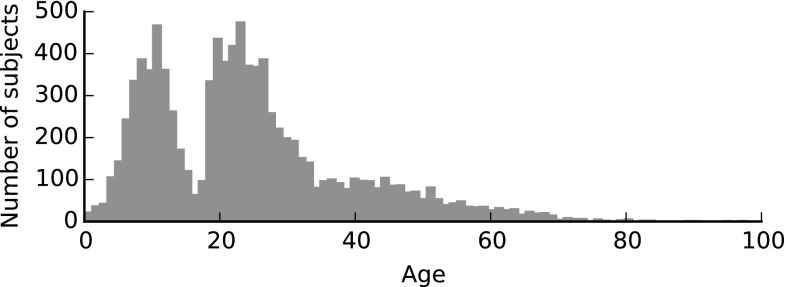
Distribution of ages of subjects included in the MeIn3D dataset

Therefore, the normalized shape parameters $\frac{\alpha _1}{\sqrt{\lambda _1}},\ldots ,\frac{\alpha _d}{\sqrt{\lambda _d}}$ are independent and identically distributed following a zero-mean and unit-variance Gaussian distribution and their squared sum, which can be written as:

8 $$\begin{aligned} F(\varvec{\alpha }) = \sum _{i=1}^{d} \frac{\alpha _i^2}{\lambda _d} \end{aligned}$$

follows a chi-square distribution with *d* degrees of freedom (Patel and Smith 2009). The above sum is actually a weighted norm of the shape vector $\varvec{\alpha }$ and yields a squared Mahalanobis distance between the current shape and the mean shape. This can be used as a measure of plausibility of the shape with shape parameters $\varvec{\alpha }$, under the current PCA model (Fig. 7).

Based on the aforementioned remarks, for every training face mesh that has been put in correspondence using NICP and afterwards subjected in Procrustes alignment, we find its shape parameters $\varvec{\alpha }$ by projecting on the initial global PCA model. Then, we use the squared norm $F(\varvec{\alpha })$ as the criterion to detect failures of the dense correspondence estimation. This is due to the fact that these failures behave as outliers of the Gaussian distribution.

We classify as outliers all shape vectors $\varvec{\alpha }$ with a squared norm $F(\varvec{\alpha })$ above a threshold $\theta _f$. This threshold is selected so that $F(\varvec{\alpha })$ is expected to be less than $\theta _f$ with a very high probability $p_f$ (e.g. 99%), under the assumed Gaussian distribution. Consequently, $\theta _f$ is specified by the evaluation of the chi-square inverse cumulative distribution function at the probability $p_f$. Note that the set of shape vectors $\alpha$ with $F(\varvec{\alpha })<\theta _f$ corresponds to a hyper-ellipse in the *d*-dimensional space of shape parameters. Following the aforementioned procedure, we find that less than 1% of the training meshes are classified as outliers.

Finally, we derive the LSFM models by applying PCA again on the corresponding training sets, after excluding the shape vectors that have been classified as outliers.

## Experiments

**Fig. 8:**
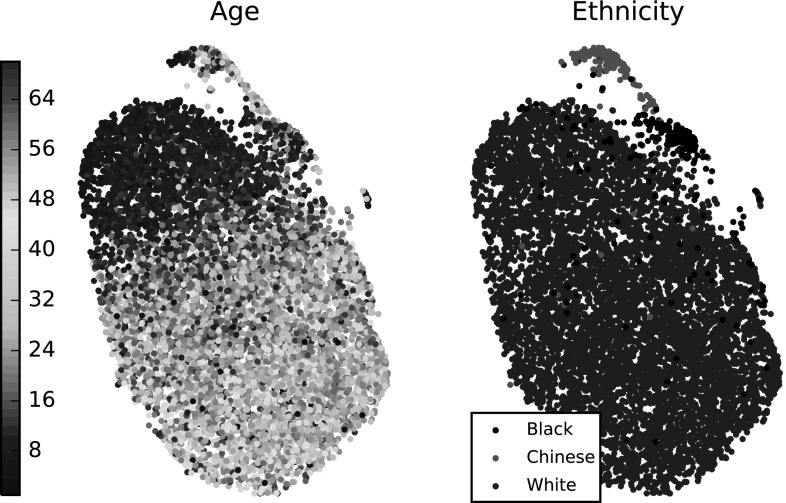
t-SNE embedding of the high dimensional face manifold in two dimensions. *Left* a clear trend of increasing age can be seen. *Right* the two smaller structures are explained largely as ethnic variations

In this section we will analyze in detail the pipeline put forward in Sect. 6. We will be applying the methodology to the newly introduced MeIn3D database, and reporting on the performance of the resultant 3DMM against three state of the art 3DMMs (Fig. 8)

### Global LSFM Model

We derive our global LSFM model (hereafter referred to as *LSFM-global*) by applying the proposed construction pipeline on the MeIn3D dataset. Figure 3 visualizes the shape component of LSFM-global by showing the mean shape along with the top five principal components of shape variation. We observe that the principal modes of variation capture trends of facial shape deformation due to gender, age, ethnicity and other variability in a particularly plausible way, yielding high-quality 3D facial shapes. Similarly, Fig. 4 visualizes LSFM-global by showing the mean texture along with the top five principal components of texture variation, all visualized on the mean shape and again clear variations in ethnicity, gender and age are visible. We observe that the textures corresponding to the mean texture and the principal components are highly-detailed and correspond to a plausible representation of texture in human faces.

An additional visualization of LSFM-global is provided by Fig. 1, which shows synthetic facial shapes generated by the model. More precisely, the shapes are synthesized using Eq. (1) with shape parameters $\alpha _i$ that are randomly sampled, assuming statistical independence and zero-mean gaussian distribution for each parameter, with variance given by the corresponding PCA eigenvalue. It can be seen that all synthetic faces exhibit a high degree of realism, including fine details in the facial structures. Furthermore, we observe that the statistical distribution of LSFM-global succeeds in capturing a plethora of demographic characteristics (age, gender and ethnicity).

### LSFM-Global Facial Manifold Visualisation

Here, we explore the properties of the LSFM-global manifold. After establishing dense correspondences with our pipeline and excluding the outliers, every retained training sample $\mathbf {X}$ is projected on the LSFM-global model and represented by the vector of shape parameters $\alpha$ that yields the closest shape within the model subspace, see Eq. (3). We then apply t-SNE (Maaten and Hinton 2008) to the shape vectors from all training samples to visualize the manifold of training shapes, as represented in the *d*-dimensional model subspace. Leveraging the per-subject demographic data we have, we are able to label samples in this space by their age, see Fig. 8 (left). Interestingly, a clear trend of increasing age across the bulk of the manifold can be seen, suggesting that the facial manifold has age-related structure.

Furthermore, we visualize the space by ethnicity, Fig. 8 (right). Note that we chose three ethnic groups for which the number of samples in the used dataset was sufficient for our analysis. We observe that t-SNE has produced a nonlinear 2D embedding that dedicates the largest area for the White ethnic group, which is not surprising, given the fact that this ethnic group is over-represented in the MeIn3D database ($82\%$ of the samples). What is particularly interesting is the fact that the clusters that are clearly separable from the main manifold are actually specific ethnic groups.

### Bespoke Demographic Models

**Fig. 9:**
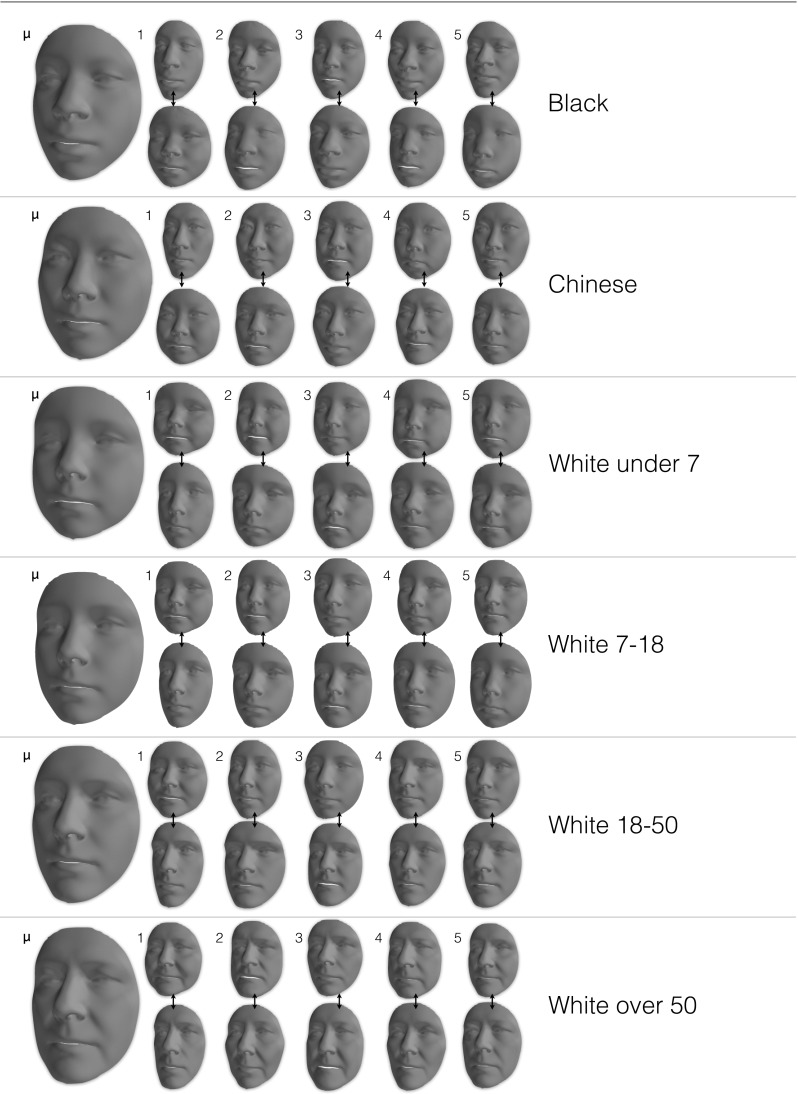
Bespoke shape models produced for specific subsets of the *MeIn3D* dataset. For each bespoke model, the figure shows the mean shape $\mu$ as well as the first five shape eigenvectors, each visualized as additions and subtractions away from the mean. In more detail, the *top* (*bottom*) row corresponds to deviating from $\mu$ in the direction of the corresponding shape eigenvector, with a weight of 3$\sigma _i$ ($-3\sigma _i$), where $\sigma _i$ is the standard deviation of the corresponding component

**Fig. 10:**
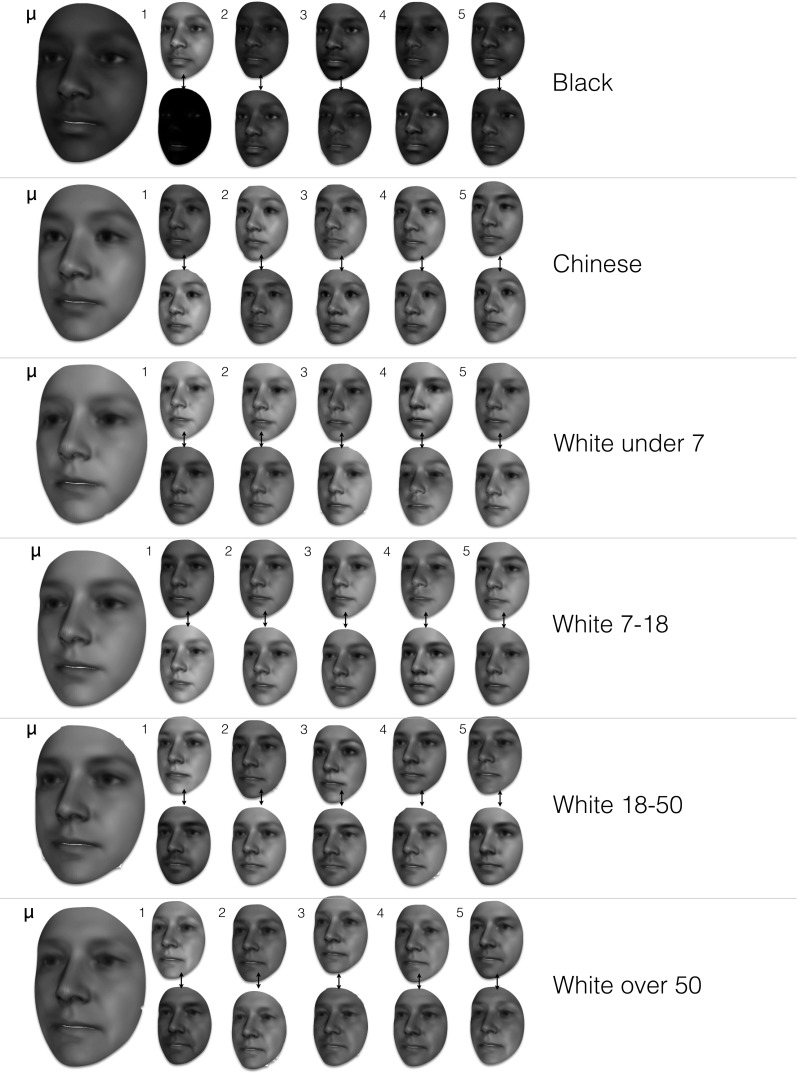
Bespoke texture models produced for specific subsets of the *MeIn3D* dataset. For each bespoke model, the figure shows the mean texture $\mu$ as well as the first five texture eigenvectors, each visualized as additions and subtractions away from the mean. In more detail, the *top* (*bottom*) row corresponds to deviating from $\mu$ in the direction of the corresponding texture eigenvector, with a weight of 3$\sigma _i$ ($-3\sigma _i$), where $\sigma _i$ is the standard deviation of the corresponding component. All textures are visualized on the mean 3D shape

These visualizations provide insight into how different regions of the high-dimensional space of human face shape and texture are naturally related to different demographic characteristics. We use this insight to define specific *bespoke models* that are trained on dedicated subsets of the full MeIn3D training population. Taking also into account the demographics of the training data available (see Sect. 4.2), we define the following groups: **Black** (all ages), **Chinese** (all ages) and White ethnic group, which due to large availability of training samples, is further clustered into four age groups: under 7 years old (**White-under 7**), 7–18 years old (**White-7 to 18**), 18–50 years old (**White-18 to 50**) and over 50 years old (**White-over 50**). The mean and most significant 5 shape components of the 6 demographic-specific models are given in Fig. 9. Likewise, Fig. 10 shows the mean and most significant 5 texture components of the six demographic-specific models visualized on the mean shape.

We combine these bespoke models in a large mixture model, which we call LSFM-bespoke. The intrinsic characteristics of both LSFM-global and LSFM-bespoke will be evaluated in the next section.

### Training and Test Sets

For all the subsequent experiments, MeIn3D dataset was split into a training set and a test set. In more detail, a set of 400 meshes of MeIn3D was excluded from the original training set to form a test set. This test set was randomly chosen within demographic constraints to ensure a gender, ethnic and age diversity. Table 1 shows the makeup of the test set. Despite the fact that this test set does not capture the full range of diversity present in the demographics of humans, its diversity is still a huge step up from existing datasets used in testing 3DMMs. Note that for the sake of fairness of the following evaluations, LSFM-global and LSFM-bespoke models were re-trained using the resultant training set. This is slightly smaller than the original training set, which included the whole MeIn3D.

### Intrinsic Evaluation of LSFM Models

Following common practice in the literature of statistical shape models, we evaluate the intrinsic characteristics of LSFM-global and LSFM-bespoke using *compactness*, *generalization* and *specificity*, see e.g. Davies et al. (2008), Brunton et al. (2014b), Bolkart and Wuhrer (2015). We consider the 3D shapes of MeIn3D dataset after establishing dense correspondences, using our pipeline.

**Fig. 11:**
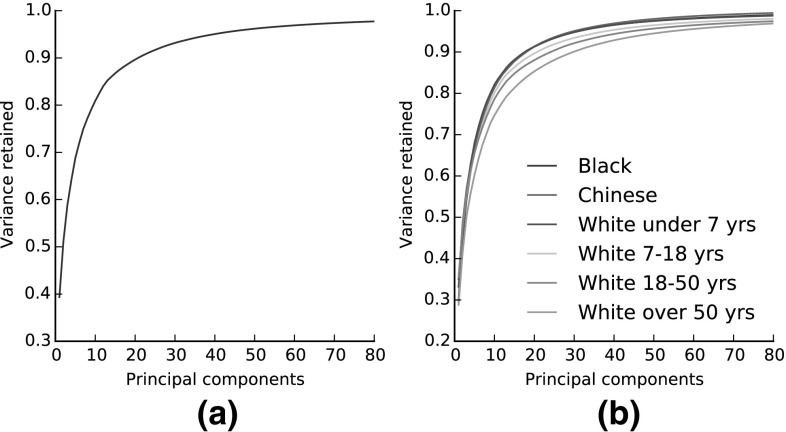
Compactness of the LSFM models. **a** LSFM-global. **b** LSFM-bespoke

Figure 11 shows the **compactness** plots for the LSFM models. Compactness measures the percentage of variance of the training data that is explained by a model when certain number of principal components are retained. Note that in the case of the bespoke models, the training samples of the corresponding demographic group are only considered, which means that the total variance is different for every model. We observe that all trained models exhibit similar traits in the variance captured, although this naturally varies with the size of the training set in each case of the tailored models. Both global and bespoke LSFM models can be considered sufficiently compact; for example for all the models, as few as 40 principal components are able to explain more than 90% of the variance in the training set.

**Fig. 12:**
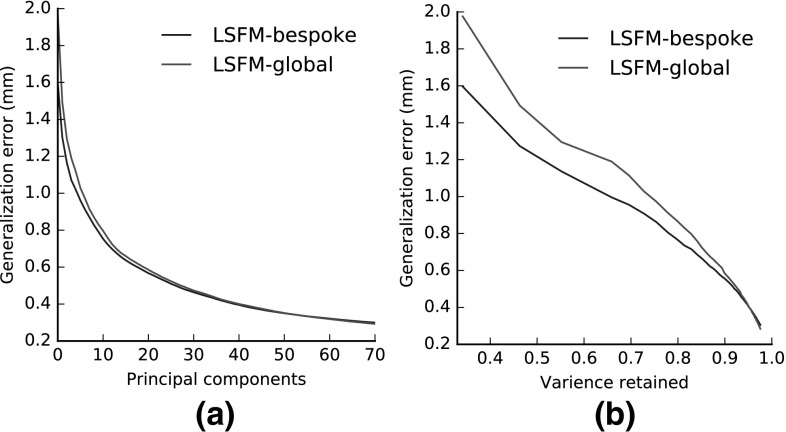
Generalization of the LSFM models

Figure 12 presents plots of model **generalization**, which measures the ability of a model to represent novel instances of face shapes that are unseen during training. To compute the generalization error of a model for a given number of principal components retained, we compute the per-vertex Euclidean distance between every sample of the test set $\mathbf {X}$ and its corresponding model projection $P(\mathbf {X})$, Eq. (3), and then take the average value over all vertices and all test samples. In order to derive an overall generalization measure for LSFM-bespoke, for every test sample we use its demographic information and project on the subspace of the corresponding bespoke model and then compute an overall average error. The number of components retained in the case of the LSFM-bespoke model is the number of components retained for the demographically-matching bespoke model for a given training sample. We plot the generalization errors with respect to both the number of principal components (Fig. 12a) and percentage of total variance retained (Fig. 12b). We observe that both LSFM-global and LSFM-bespoke are able to generalize well, since for even low number of components and total variance retained, they yield particularly low generalization errors. Interestingly, we see in Fig. 12a that LSFM-bespoke achieves superior generalization measures when compared to LSFM-global for an equivalent number of components for fewer than 60 components. After this stage the global model starts to outperform the specific models, which might attributed to the fact that many of the specific models are built from smaller cohorts of training data, and so run out of interesting statistical variance at an earlier stage. When changing the visualization to one based on retained variance (Fig. 12b), we observe that the demographic-specific LSFM-bespoke model achieves better generalization performance for the vast majority of values of retained variance.

**Table 1:** Proportions of each demographic group represented in the MeIn3D test set

Demographic	Count
Black	40 (20 male and 20 female)
Chinese	40 (20 male and 20 female)
White-under 7	80 (40 male and 40 female)
White-7 to 18	80 (40 male and 40 female)
White-18 to 50	80 (40 male and 40 female)
White-over 50	80 (40 male and 40 female)

**Fig. 13:**
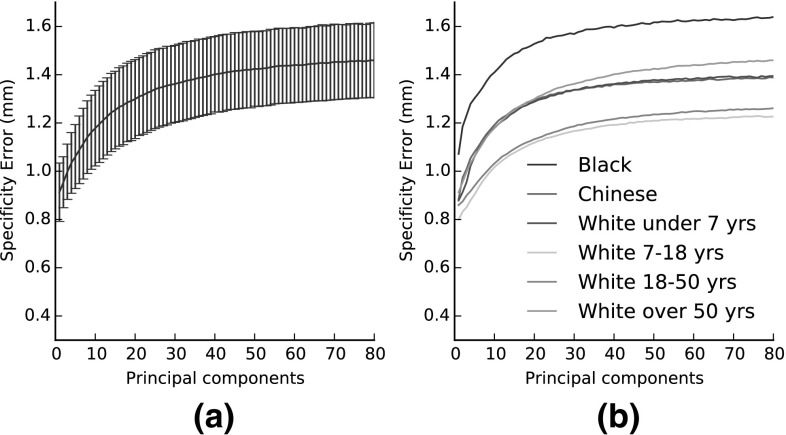
Specificity for each of the tailored models. **a** specificity and standard deviation for global model. **b** specificity for the tailored models

Figure 13 presents the **specificity** of the introduced models, which evaluate the validity of synthetic faces generated by a model. To measure this, we randomly synthesize 10,000 faces from each model for a fixed number of components and measure how close they are to the real faces of the test set. More precisely, for every random synthetic face, we find its nearest neighbor in the test set, in terms of minimum (over all samples of the test set) of the average (over all vertices) per-vertex distance. We record the mean of this distance over all samples as the specificity error. Figure 13a contains the specificity plot for LSFM-global (mean value as well as standard deviation bars), whereas Figure 13b contains the specificity plots for all models of LSFM-bespoke (mean values only; the standard deviation bars have been omitted for the sake of visualization clarity). We observe that in all the cases, the specificity errors attain particularly low values, in the range of 1–1.6 mm, even for a relatively large number of principal components. This is a quantitative evidence that the synthetic faces generated by both global and bespoke LSFM models are realistic, which complements the qualitative observations of Sect. 7.1. Interestingly, Fig. 13b suggests that specificity error is larger for models trained from smaller populations, as e.g. in the case of Black model. Apart from the lack of sufficient representative training data, this might also be attributed to the fact that the space of such models is more sparsely populated by training samples, so the nearest neighbor error tends to be larger, as compared to other models with more training data. Furthermore, it can be seen that the lowest specificity error comes from the White-7 to 18 model, which is trained on a large number of samples that lie on a smaller cluster of the space, leading to a highly specific model.

### Comparison of Dense Correspondence Methods

We now repeat select studies from the previous sections (using the same parameters as before), only now we vary the dense correspondence algorithm employed (all models are built using data from the global MeIn3D dataset). With this work, we will empirically motivate our choice of NICP for providing dense correspondences in our method over the alternatives (UV-OF, UV-TPS).

Note that for UV-OF, we used the Improved TV-L1 algorithm (Wedel et al. 2009), which is a relatively recent state-of-the-art optical flow algorithm. This algorithm demonstrates improved robustness and accuracy, as compared to traditional optical flow methods.

Figure 5 shows an example dense correspondence result for NICP, UV-TPS and UV-OF. Most striking is that NICP is better able to deal with a larger region of the facial surface. The UV-based techniques cannot interpolate well for broader regions of the head as areas like the underside of the chin and the interior of the mouth are not well mapped onto a cylinder. Furthermore, NICP has some hole filling capability, where the natural result of the optimization problem leads to missing regions of the target being replaced by interpolated values drawn from the corresponding part of the template (we refer the interested reader to Amberg et al. (2007) for details). In this example this infilling can be seen to successfully recover the chin region, which is entirely missing in the original scan.

**Fig. 14:**
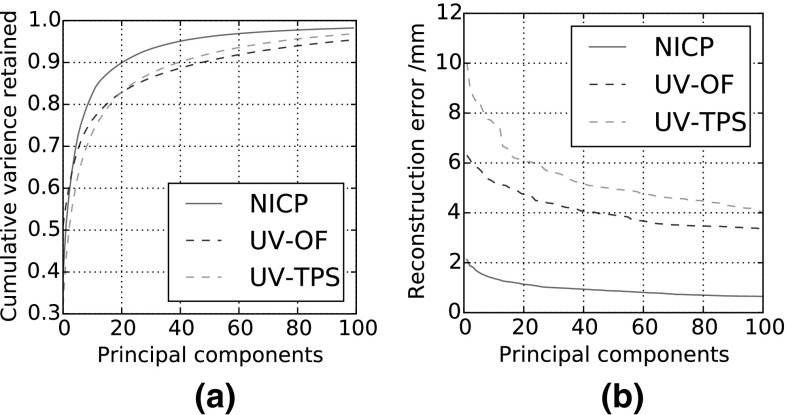
**a** Compactness for LSFM models built with differing dense correspondence methods. **b** Mean dense point-to-point reconstruction error when reconstructing out-of-sample faces (drawn from BU3D-FE) in the LSFM shape models

Figure 14a shows how NICP-based correspondences generate a model with a superior compactness quality. Figure 14b reports the mean dense reconstruction error for out-of-sample BU3D-FE faces with the different dense correspondence techniques for varying retained parameters. This is perhaps the most direct measure we have presented so far of 3DMM performance, and in this experiment we see that NICP has produced a far more useful basis in our particular context.

### Fitting Application

In order to gauge the quality of the LSFM-global model in comparison with the state-of-the-art, we evaluate the performance of the models in a real-world fitting scenario. We compare with three publicly available Morphable Models of human faces in neutral expression, namely the *BFM model* (Paysan et al. 2009a, Paysan et al. 2009b), the PCA model of Brunton et al. (2014b), Bolkart et al. (2013), which will be hereafter referred to as *Brunton et al. model*, and the Surrey face model (Huber et al. 2016). Note that for the sake of fairness towards the existing models, we do not consider the bespoke LSFM models in the fitting experiment, since these models use additional information related to demographics.

**Fig. 15:**
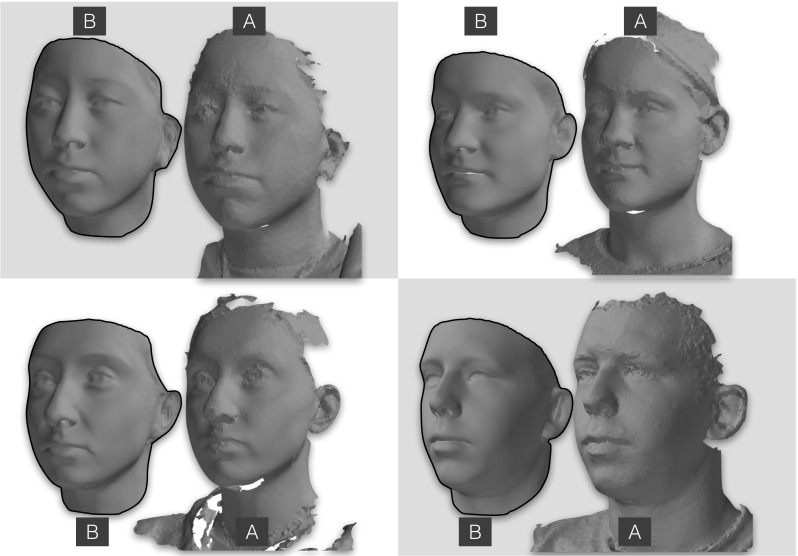
Four examples of reconstructions performed using the LSFM-global model on individuals from the BU3D-FE database. For each individual, **a** is the original scan, and **b** is the reconstruction attained

Note that for all versions of LSFM-global evaluated hereafter, we choose the number of principal components, so as to explain $99.5\%$ of the training set variance. For BFM, Brunton et al. and Surrey models, we use all the principal components, as given by the publicly available versions of these models.

To evaluate the fitting performance of every tested model, every mesh in the adopted test set is automatically annotated with facial landmarks using our technique outlined in Sect. 6.1. The same set of landmarks is manually placed on the mean faces of every model, and subsequently used to similarity-align them with every mesh of the test set. Similarly to Brunton et al. (2014b), Zulqarnain Gilani et al. (2015), a simple model fitting is employed, which consists of

1. Search for the nearest vertex in the test mesh to establish correspondences between that mesh and the model
2. Project the test mesh onto the model using Eq. (3).

The per-vertex fitting error is then computed as the distance between every vertex of the test mesh and the nearest-neighbor vertex of the corresponding model-based fitting. Note that we use a simple fitting strategy to provide an appropriate mechanism to benchmark models against one another fairly—the fitting algorithm itself is not under test here, but rather the models themselves.

We evaluate a dense surface error for vertices of the raw MeIn3D scans of the test set, to remain fair across the different model densities. Furthermore we only consider the vertices within a central region of the face, which is certainly present in all models under evaluation. This means that any differences present between different models (throat, ears, inner mouth) do not come into play. Given that we evaluate on raw scans without considering any dense correspondence estimation, we lack the dense semantic understanding of the face. In the absence of this, we chose the vertices that we evaluate on by using a fixed radial distance from the (annotated) nosetip of each MeIn3D scan in the test set. Only vertices within this region, which is a tight crop of the inner facial features, are considered in our error metric.

**Fig. 16:**
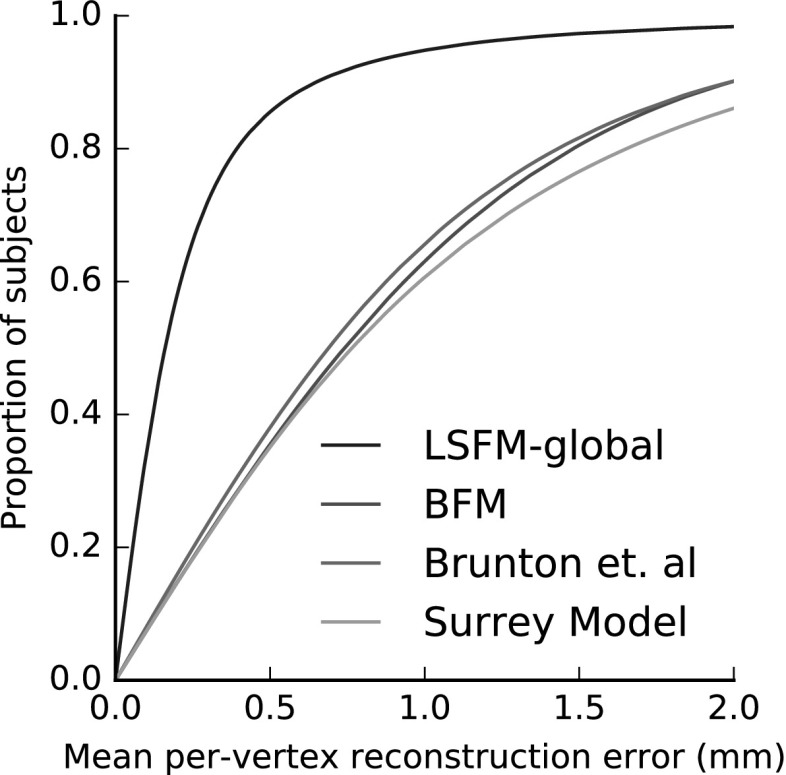
Cumulative error distributions of the per-vertex fitting error for the publicly-available models under test

Figure 16 compares the fitting performance of LSFM-global against BFM, Brunton et al. and Surrey models, in terms of cumulative error distribution (CED) curves of per-vertex fitting errors. We observe that LSFM-global achieves exceptionally improved accuracy and robustness, as compared to the other two models. This is attributed to the larger training sample used, the increased demographic range, and the quality of the MeIn3D scans. We will explore the dimorphic and quantity effects on the model performance in Sect. 7.8. We also note that this is the first time that existing models are evaluated against a dataset containing a large variation in ethnicity and age. The significantly larger variability in the training set of LSFM-global allows it to generalize well to a much wider variety of faces than the more narrowly-focused existing models. We provide visualizations of fittings for four subjects from BU3D-FE from the LSFM-global model in Fig. 15.

**Fig. 17:**
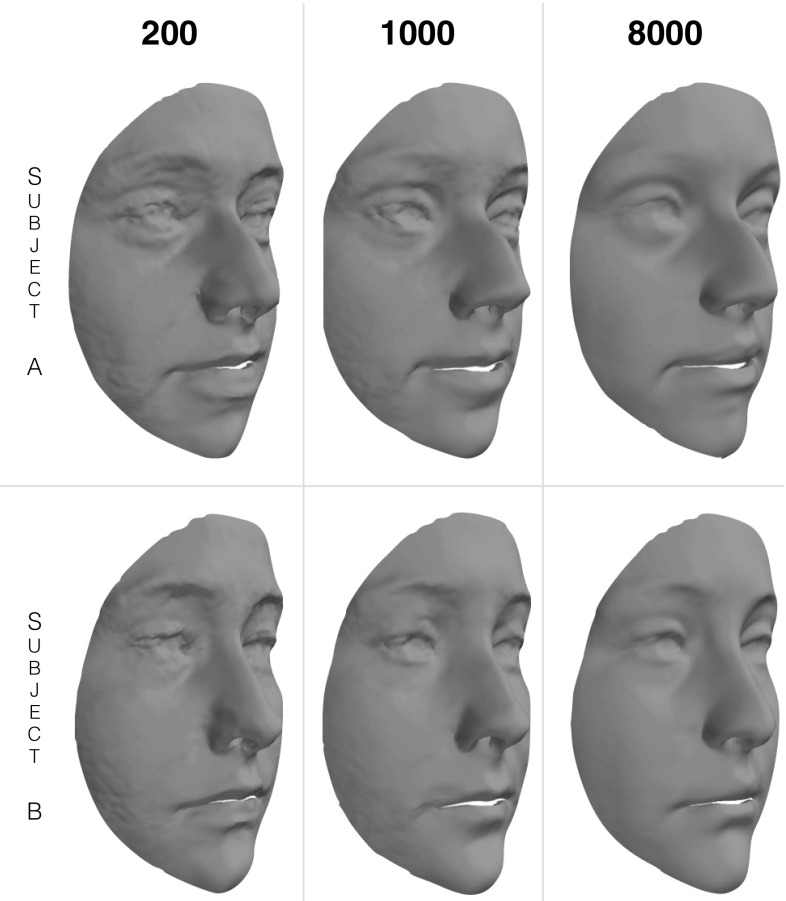
Two examples of out of sample reconstructions from BU3D-FE using LSFM models trained from 200, 1000, and 8000 subjects

**Fig. 18:**
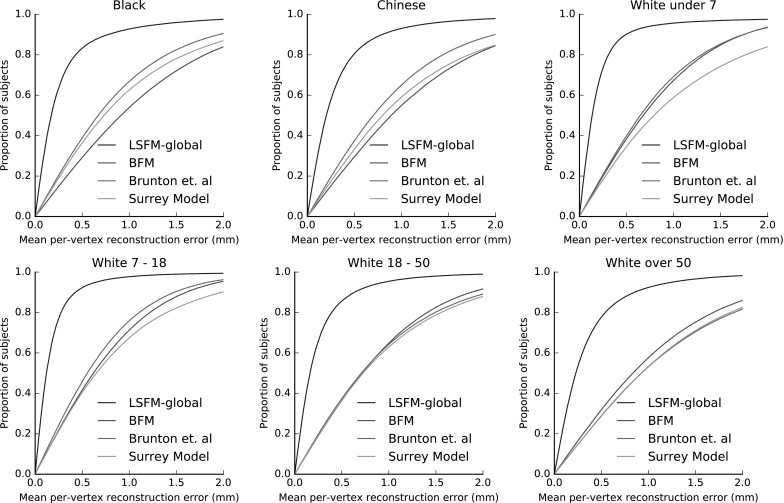
Fitting results broken down by different demographic groups

### Effect of Demographics and Training Set Size

MeIn3D is simultaneously the largest and most variable 3D facial dataset that has existed to date. To provide greater insight into how demographic variability and training set size impact the performance of 3D Morphable Models, we now explore in detail the impact of these two factors on the intrinsics and fitting application of our model (Figs. 16, 17).

#### Demographics-Specific Analysis of 3D Model Comparisons

In this section, we present a more detailed view of the 3D model fitting comparisons of Sect. 7.7. We report performance measures of the compared models on every considered demographic group separately. Figure 18 presents the CED curves of per-vertex fitting error of all compared models for each considered demographic group. Interestingly, Brunton et al. model outperforms BFM in all groups except for the group White-over 50, where the situation is clearly reversed. Also, Surrey model performs worse than BFM on the groups of White ethnicity, but on contrary it has a clear advantage over BFM on Black and Chinese groups. Finally, LSFM-global clearly outperforms all other models in all groups, even in groups that are very similar to the demographics of the training data that the other models have built upon, such as the group White-18 to 50.

**Fig. 19:**
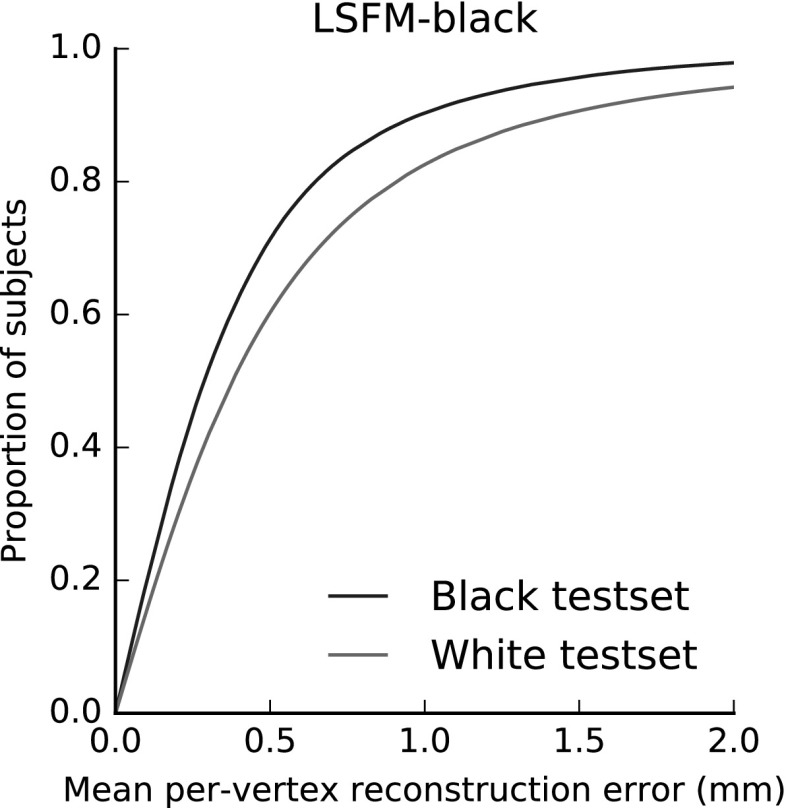
In this experiment, a model trained from samples purely drawn from a Black ethnic group is fitted to both a demographically similar Black test set and to an ethnically different white database. The performance is optimal when the demographics of the model match that of the test set

Intuition suggests that bespoke facial models have value in providing a tailored, more compact model to fit out of sample data. To explore this is indeed the case quantitatively, we construct a model from one demographic group (Black) and perform the a fitting against (a) an ethnicity matched test set and (b) a non-ethnicity matched test set (combination of all White test sets). Figure 19 shows the result of this test. The same model can clearly be seen to perform better on the demographically matched test set, demonstrating the significance of demographics in 3D facial modelling, and the value of bespoke demographic facial models.

**Fig. 20:**
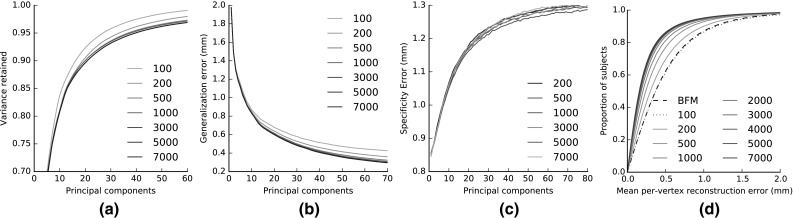
Effect of training set size on odel intrinsics. **a** Compactness. **b** Generalization. **c** Specificity, and on the fitting performance of the model **(d)**

#### Effect of Training Set Size

Given the fact that MeIn3D dataset is so much larger than existing training data sets, it is natural to question the effect of varying the size of the training set on the performance of the constructed 3DMM. To explore this, we repeat the intrinsic evaluation of Sect. 7.5 as well as the fitting experiment of Sect. 7.7 for different versions of the LSFM-global model, trained from varying numbers of samples.

The results are visualized in the plots of Fig. 20. Regarding the intrinsic evaluation, we first of all observe that the compactness curve goes down as the training size is increased. This is an expected artifact because the compactness measure gives a negative bias to the cases of larger training sets, since the total variance increases significantly. However, this does not mean that the real compactness of the model becomes worse. In addition, we observe that the generalization error decreases significantly as the training size increases. This is attributed to the fact that the statistical model can generalize better when it has been learnt from more training samples. In addition, it is interesting to notice that the specificity measures do not exhibit any statistically significant change with the size of the training set, with the corresponding curves being very close with each other. This means that according to that measure, the faces synthesized by the model retain their degree of realism as the training size increases. But in the same time, they seem to be able to represent a wider variety of human faces, as the aforementioned results on generalization suggest.

Regarding the model fitting performance (Fig. 20d), we can see clear improvements for around one order of magnitude more data than is currently used, albeit with diminishing returns beyond a few thousand samples. We also note that even with only 100 samples, LSFM-global matches the performance of the BFM, which was trained on 200 samples. This can be attributed to the larger variability of the LSFM training set, demonstrating how crucial this is for building effective 3DMMs.

Finally, Fig. 17 visually shows the effect on two BU3D-FE subject reconstructions for models trained from varying numbers of samples. As the training size increases, the model stops overfitting to noise present in the raw scans, and starts to capture the actual shape of the individual more accurately.

#### Limiting Both the Demographics Variability and the Training Size

In the extensive experiments of the previous sections, we have seen that our model yields a significantly improved performance as compared to the existing publicly available 3D face models, both in terms of quantitative and qualitative evaluation. However, it has not been clear until now what is the merit of our 3DMM construction pipeline on this success. Therefore, in this section we evaluate our pipeline by factoring out the advantages that our large-scale dataset offers us.

In more detail, we apply our pipeline on a conventional, small-scale dataset (200 random samples from MeIn3D that correspond to the White ethnic group), which has the same size and similar demographics to that used in the BFM model. The resultant model, which we call SSM-200W (small scale model, built on 200 White subjects), serves only the evaluation purposes of this section and is obviously not to be preferred over our LSFM models.

**Fig. 21:**
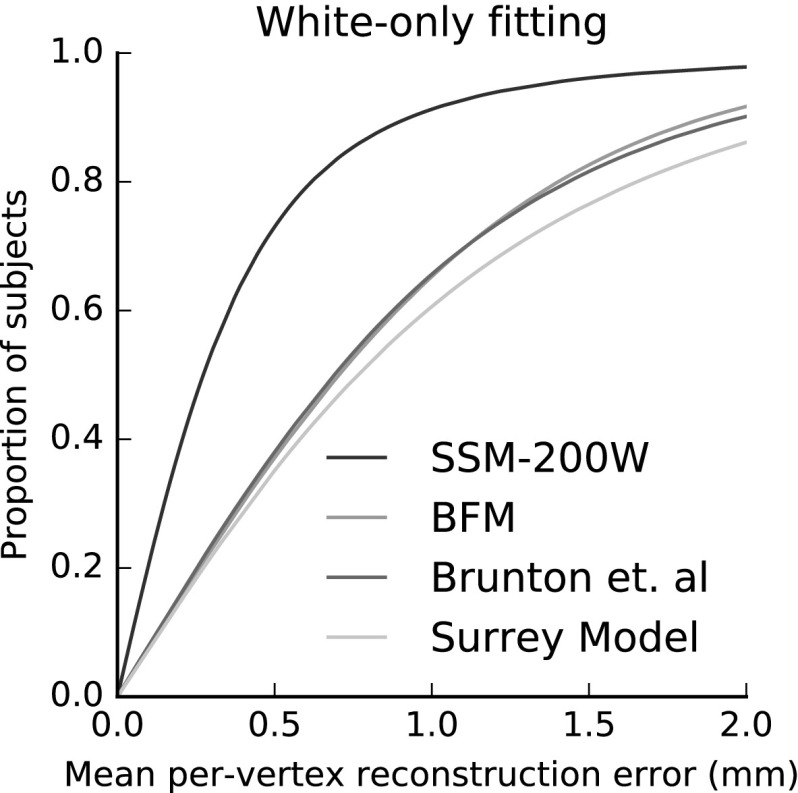
In this experiment, we evaluate our model construction pipeline by applying it to a small-scale training set of 200 white subjects, similar to the one used in the training of BFM. We compare the resultant model (SSM-200W) with the publicly available models, by fitting all models to a test set from the white group. Cumulative error distribution curves of the per-vertex fitting error are plotted for each tested model

We then compare SSM-200W with the existing models on a test set (disjoint from the training set) that also contains samples from the White ethnic group only. This compares solely our model building procedure with the corresponding procedures of the existing models. For this comparison, we follow again the model fitting evaluation protocol that we described in Sect.7.7. Figure 21 presents the results, in terms of CED curves of per-vertex fitting errors. We observe that SSM-200W clearly outperforms the existing 3D facial shape models. This clearly shows the effectiveness and robustness of our model building pipeline.

### Age Classification From 3D Shape

As a final evaluation, we use the unique traits of the MeIn3D dataset to compare the descriptive power of LSFM-global, BFM and Brunton et al. models in an age classification experiment. In more detail, we project all the face meshes of the training set onto each of the four models and use the corresponding shape vectors, $\varvec{\alpha }$, to represent them, see Eq. (3). Using the demographic information of MeIn3D dataset, we train a linear support vector machine classifier for each model, which maps the corresponding shape vectors to four age classes: under 7, 7–18, 18–50, over 50.

To measure the classification accuracy, we project all samples from the test set onto the models and then use the classifier to predict the age bracket for the test subjects. This provides an application-oriented evaluation of the quality of the low-dimensional representation that each 3DMM provides for the large variety of faces present in LSFM. As can be seen in Table 2, the LSFM-global model outperformed existing models in precision and recall and f-score, correctly classifying the age of 74% of the subjects in the challenging test set .

**Table 2:** Mean age classification scores

	Precision	Recall	F-Score
LSFM-global	**0.74**	**0.61**	**0.60**
BFM	0.71	0.54	0.51
Brunton et al.	0.68	0.53	0.52
Surrey Model	0.70	0.44	0.39

Bold values indicate the best performance in each metric

## Conclusions and Future Work

We have presented LSFM, the most powerful and statistically descriptive 3DMM ever constructed. By making both the LSFM software pipeline and models available, we help to usher in an exciting new era of large scale 3DMMs, where construction is radically simpler and large-scale models can become commonplace. We have demonstrated that our automatically constructed model comfortably outperforms existing state of the art 3DMMs thanks to the sheer variety of facial appearance it was trained on, and further reported on how the size of 3D datasets impacts model performance. We have explored for the very first time the structure of the high dimensional facial manifold, revealing how it is clustered by age and ethnicity variations, and demonstrated for the first time accurate age prediction from 3D shape alone. The ability of the model to differentiate faces according to ethnicity suggests that it is sensitive to subtle genetic variation. This raises the possibility that it may be useful in future medical work, for instance providing the basis for an automated diagnostic tool for patients with genetic conditions. In future work we will analyze in detail the qualities of the LSFM model, exploring what it can tell us about human face variation on the large scale, as well as exploring novel statistical methods for large-scale 3DMM construction. We will furthermore explore how shape and texture information can be fused in dense correspondence approaches in order to maximise the accuracy of the registration of facial meshes.

## Footnotes

1 Apart from soft-tissue facial shape, 3D morphable models have also been successfully applied in the modelling of human skull shape, see e.g. Paysan et al. (2009c), Duan et al. (2015), Staal et al. (2015).

2
http://faces.cs.unibas.ch/bfm/.

3
http://statistical-face-models.mmci.uni-saarland.de/.

4
http://cvssp.org/faceweb/3dmm/facemodels/.

5
https://github.com/menpo/lsfm.

6
http://www.ibug.doc.ic.ac.uk/resources/lsfm.
